# Is HIV-1 infection associated with endothelial dysfunction in a population of African ancestry in South Africa?

**DOI:** 10.5830/CVJA-2010-056

**Published:** 2011-06

**Authors:** C Fourie, J Van Rooyen, A Schutte, M Pieters, K Conradie, T Hoekstra

**Affiliations:** HART (Hypertension in Africa Research Team), Physiology, North-West University, Potchefstroom, South Africa; HART (Hypertension in Africa Research Team), Physiology, North-West University, Potchefstroom, South Africa; HART (Hypertension in Africa Research Team), Physiology, North-West University, Potchefstroom, South Africa; TReNDS Centre of Excellence – Nutrition, North West University, Potchefstroom, South Africa; TReNDS Centre of Excellence – Nutrition, North West University, Potchefstroom, South Africa; Julius Centre for Health Sciences and Primary Care, University Medical Centre Utrecht, Utrecht, the Netherlands

**Keywords:** HIV-1, South Africa, endothelial dysfunction, vascular aging, never treated, inflammation

## Abstract

**Abstract:**

The chronic infection status suffered by HIV-infected individuals promotes chronic arterial inflammation and injury, which leads to dysfunction of the endothelium, atherosclerosis and thrombosis. Although HIV-1 subtype C is prevalent in South Africa and accounts for almost a third of the infections worldwide, this subtype differs genetically from HIV-1 subtype B on which the majority of studies have been done. The objective of this study was to assess whether newly identified, never-treated, HIV-1-infected South African participants showed signs of endothelial dysfunction, accelerated atherosclerosis and increased blood coagulation.

We compared 300 newly diagnosed (never antiretroviral-treated) HIV-infected participants to 300 age-, gender-, body mass index- and locality-matched uninfected controls. Levels of high-density lipoprotein cholesterol (HDL-C), triglycerides, interleukin-6 (IL-6), C-reactive protein (CRP), intercellular adhesion molecule-1 (ICAM-1), vascular cell adhesion molecule-1 (VCAM-1), fibrinogen and plasminogen activator inhibitor-1 (PAI-1), and carotid radialis pulse wave velocity (cr-PWV) were determined. The HIV-infected participants showed lower HDL-C and higher IL-6, CRP, ICAM-1 and VCAM-1 levels compared to the uninfected controls. No differences in fibrinogen and PAI-1 levels were detected. A continuous positive trend of increasing age with cr-PWV was detected in the HIV-infected group.

Our findings suggest inflammatory injury of the endothelium, pointing to endothelial dysfunction of never-treated HIV-1-infected South Africans of African ancestry. Although no indication of a prothrombotic state could be detected, there was an indication of accelerated vascular aging and probable early atherosclerosis in the older HIV-infected participants.

## Abstract

Several cardiovascular risk factors have been associated with or seen in the human immunodeficiency virus (HIV)-infected population since the trend of longer life expectancy due to antiretroviral (ARV) therapy.^1^,^2^ Worldwide, various forms of cardiovascular involvement, such as endothelial dysfunction,^3^ accelerated atherosclerosis^4^ and coagulation disorders^5^ have been documented among HIV-infected individuals. In South Africa, atherosclerotic disease, historically not common in most black Africans, is increasing.^6^ One of the important contributors, at least in part, to this increase could be the cardiac complications related to HIV infection, as South Africa is the country with the highest number of HIV infections in the world.^7^

The chronic infection of HIV-infected individuals promotes chronic arterial inflammation and injury, which in turn, promotes dysfunction of the endothelium, atherosclerosis and thrombosis.^5^,^8^ Endothelial injury and dysfunction have been proposed as plausible links between HIV infection and atherosclerosis.^9^ The development of atherosclerosis may be the consequence of infection-triggered endothelial damage,^10^ and atherosclerotic cardiovascular events are commonly manifested via thrombotic events.^11^

Increased levels of the inflammatory markers C-reactive protein (CRP), interleukin 6 (IL-6),^8^ and cell adhesion molecules, intercellular adhesion molecule-1 (ICAM-1) and vascular cell adhesion molecule-1 (VCAM-1)^3^ have been reported in the HIV-infected population.^2^,^12^ Accelerated atherosclerosis has also been detected in HIV-infected patients,^1^,^13^ and a wide range of coagulation disorders may be associated with HIV infection itself.^5^

Although an estimated 5.5 million people are living with HIV in South Africa^7^ where HIV-1 subtype C prevails,^14^,^15^ the majority of studies on HIV have been done on HIV-1 subtype B, which is responsible for infections in North America, Europe and Australia.^15^,^16^ Subtype C accounts for 55 to 60% of all HIV infections worldwide and differs as much as 30% in its genome from subtype B.^17^,^18^ The clinical consequences of subtype variations are still unknown and the effect of the HIV-1 subtype C virus on the vascular system is not certain.

Recently it has been recommended that HIV infection *per se* should count as a coronary risk factor, similar to the traditional cardiovascular risk factors (smoking, hypertension, hypercholesterolaemia and diabetes).^4^ Although data of Lorenz *et al*. support the hypothesis that HIV infection promotes early atherosclerosis independently of the ‘classical’ vascular risk factors,^13^ the role of HIV infection as a risk factor for premature atherosclerosis is still controversial.^9^,^19^ There is also uncertainty about the relative contribution of the viral infection, the virus itself, the associated inflammatory response, antiretroviral therapy, and the interaction between them and the cardiovascular risk factors seen in the HIV-infected population.^20^,^21^

In view of the above, the aim of this study was to assess whether newly identified, never-treated, HIV-1-infected South Africans of African ancestry showed signs of endothelial dysfunction, accelerated atherosclerosis and increased coagulation, which could lead to thrombosis.

## Methods

This sub-study is nested in the larger international PURE (Prospective Urban and Rural Epidemiological) study. The international PURE study is a longitudinal, multinational study that will address questions regarding the cause and development of cardiovascular risk factors and chronic disease within populations in developing countries, including South Africa.^22^

The South African leg of the study was performed in the North West province where a total of 2 000 participants (1 000 urban and 1 000 rural) were randomly recruited from a rural and urban setting and screened during the baseline phase in 2005. The inclusion criteria were volunteers older than 35 years who were non-users of any chronic medication and with no self-reported diseases. For this case–control sub-study, 300 newly identified HIV-infected participants of the baseline PURE study population were individually matched with 300 HIV-uninfected participants, according to age, gender, body mass index (BMI) and locality (urban and rural). The protocol appropriate to this sub-study will be discussed.

All participants provided signed informed consent after all procedures were explained to them in their home language. The study protocol complies with the Declaration of Helsinki as revised in 2004,^23^ and was approved by the Ethics Committee of the North-West University, Potchefstroom, South Africa.

Permission to execute the study was obtained from the provincial Department of Health, local authorities and from the tribal chief in the rural area. Over a period of 12 weeks, 30 to 35 participants arrived at the research locality of the rural or urban areas daily at about 07:00 each morning after a 10- to 15-minute drive (provided by the research team) from their communities.

The participants were introduced to the setup and after the procedures were explained, they signed the informed consent forms and received HIV pre-counselling given by trained counsellors. The HIV status of the participants was revealed during individual post-counselling and the infected participants were referred to their local clinics or hospitals for follow up and CD_4_ cell count determination.

During the course of the morning, demographic, lifestyle and food frequency questionnaires were completed with the help of the specially trained field workers in the subjects’ home language. Lifestyle data included self-reported current tobacco use, alcohol intake as well as medical history. Height, weight, hip and waist circumference (WC) were measured (Precision Health Scale, A & D Company, Japan; Invicta Stadiometer, IP 1465, UK; Holtain unstretchable metal tape) using standardised procedures.^24^

Systolic (SBP) and diastolic blood pressure (DBP) were obtained with the validated OMRON HEM-757 device. After a 10-minute rest period, blood pressure measurements were performed twice (five minutes apart) on the right arm (brachial artery), while the participant was seated upright and relaxed with his/her right arm supported at heart level. Appropriate cuffs were used for obese participants. MAP pressure was calculated by diastolic blood pressure plus one-third of pulse pressure. Carotid-radialis pulse wave velocity (cr-PWV) was measured on the left side of each participant in the supine position, making use of the Complior SP (Artech-Medical, Pantin, France) apparatus.

Fasting blood samples were obtained from the antebrachial vein using a sterile winged infusion set and syringes. Serum and plasma were prepared according to appropriate methods and stored at –80°C in the laboratory. In the rural area, serum/plasma was stored at –18°C (no longer than five days) until it could be transported to the laboratory facility, where it was stored at –80°C until analysis.

## Biochemical analyses

Quantitative determination of high-density lipoprotein cholesterol (HDL-C), triglycerides (TG), high-sensitivity C-reactive protein (hsCRP), glucose and creatinine concentrations in the serum of the participants was done with the Konelab™ auto analyser (Thermo Scientific, Vantaa, Finland). This is a clinical chemistry analyser for colorimetric, immunoturbidimetric and ion-selective electrode measurements. Creatinine clearance rate was estimated using the Cockcroft-Gault formula.

Serum concentrations of high-sensitivity interleukin-6 (hsIL-6) were measured using human enzyme-linked immunosorbent assays (Quantikine® HS ELISA, R&D Systems, Minneapolis, USA). Concentrations of serum intercellular adhesion molecule 1 (sICAM-1) and serum vascular cell adhesion molecule 1 (VCAM-1) were assessed by sandwich ELISAs (human sICAM-1 and human sVCAM-1 assay, IBL, Hamburg, Germany).

The quantitative determination of fibrinogen in plasma was performed by the Multifibren U-test (Dade Behring), a modification of the Clauss method on the Dade Behring BCS coagulation analyser. The quantification of plasminogen activator inhibitor-1 (PAI-1) activity was performed by a chromogenic assay kit, Spectrolyse®/pL PAI-1 (Trinity Biotech plc, Bray Co, Ireland).

HIV status was determined according to the protocol of the National Department of Health of South Africa. A rapid card test, First Response (PMC Medical, India) was used for testing, using whole blood. If tested positive, the result was repeated with the Pareeshak (BHAT Bio-tech India) card test for confirmation. The HIV-1 subtype C epidemic prevalent in South Africa has been established by serotyping and genotyping.^7^,^14^,^15^ The CD_4_ cell counts were obtained from the local clinic or hospital within three months of the data collection of the baseline phase. CD_4_ cell counts could only be obtained for 72 participants, as these were the only participants who visited their local clinic or hospital for follow-up. The CD_4_ counts were determined (in whole blood) by the National Health Laboratory using flow cytometric analysis (Beckman COULTER® EPICS® XLTM, Fullerton, USA).

## Statistical analysis

All data were statistically analysed by means of Statistica v.8 (Statsoft Inc., OK, USA, 2008). Mean values, standard deviations and standard errors were calculated. The distributions of hsCRP, hsIL-6, sICAM-1, sVCAM-1, fibrinogen and PAI-1 were normalised by logarithmic transformation before analysis, reporting the geometric mean and the fifth and 95th percentile intervals. Independent *t*-tests were used to compare the uninfected group with the infected group and the group with the nadir CD_4_ cell count.

ANOVA and Tukey’s *post hoc* test for multiple comparisons were used to compare the characteristics of the continuous variables of the HIV-uninfected, -infected and nadir CD_4_ cell count groups. Chi-square tests were done to compare data of categorical variables. An analysis of covariance (ANCOVA) and Bonferoni *post hoc* test were performed to compare the inflam matory and cell adhesion biomarkers of the infected and uninfected groups while adjusting for mean arterial pressure (MAP), tobacco and alcohol use.

Partial correlations were performed in the HIV-uninfected, -infected and nadir CD_4_ cell count groups while adjusting for MAP, tobacco and alcohol use. For the cr-PWV analysis, the subjects were divided into three age groups (with 10-year intervals; group 1 ≤ 40 years, group 2 = 40–50 years and group 3 ≥ 50 years) and ANCOVA (adjusted for gender, BMI, MAP, tobacco and alcohol use) was performed. The cr-PWV was plotted for the HIV-infected and -uninfected participants.

## Results

The characteristics of the HIV-infected participants and matching controls as well as the subgroup of the HIV-infected participants with a nadir CD_4_ cell count < 200 cells/mm^3^ are reported in Table 1. Due to individual matching, age and BMI values were identical in the HIV-infected and uninfected (control) groups. The SBP and HDL-C values were lower and the TG levels, TG:HDL-C ratio, and hsIL-6, hsCRP, sICAM-1 and sVCAM-1 concentrations were higher in the HIV-infected compared to the uninfected participants. The lowest HDL-C levels were seen in the HIV-infected participants with a nadir CD_4_ cell count.

**Table 1.** Characteristics Of Participants: HIV Uninfected, HIV Infected, And HIV Infected With A Nadir CD_4_ Cell Count < 200 Cells/mm^3^

	*HIV uninfected (n = 300)*	*p-value^†^ HIV uninfected/infected*	*HIV infected (n = 300)*	*p-value^†^ HIV uninfected/nadir CD_4_ cell count*	*Nadir CD_4_ cell count (n = 18)*	*p-value^‡^ trend*
Age (years)	44.0 ± 7.81	0.971	44.0 ± 8.04	0.679	44.8 ± 8.48	0.904
Men/women (*n*)	116/184	1.000	116/184	0.135	10/8	0.316
Body mass index (kg/m^2^)	22.8 ± 5.48	0.916	22.9 ± 5.59	0.122	20.8 ± 3.96	0.258
Systolic blood pressure (mmHg)	129 ± 21.8	0.003	124 ± 21.8	0.309	124 ± 17.5	0.015
Diastolic blood pressure (mmHg)	85.9 ± 14.3	0.099	84.0 ± 14.7	0.145	80.9 ± 10.6	0.191
Mean arterial pressure (mmHg)	100 ± 16.1	0.024	97.5 ± 16.7	0.179	95.3 ± 12.1	0.179
Carotid radialis pulse wave velocity (m/s)	10.9 ± 2.30	0.606	11.1 ± 2.10	0.307	11.5 ± 2.30	0.561
Lipids:						
HDL-C (mmol/l)	1.70 ± 0.71	< 0.001	1.23 ± 0.58	< 0.001	1.07 ± 0.47	< 0.001
TG (mmol/l)	1.15 ± 0.75	0.031	1.29 ± 0.77	0.814	1.19 ± 0.76	0.085
TG:HDL-C ratio	0.86 ± 1.21	< 0.001	1.41 ± 1.47	0.006	1.19 ± 0.21	< 0.001
Inflammatory markers:						
hsIL-6 (pg/ml)	3.72 (1.11–16.9)	< 0.001	4.70 (1.29–20.9)	0.124	5.03 (1.10–22.3)	0.002
hsCRP (mg/l)	2.13 (0.23–29.2)	< 0.001	3.31 (0.32–50.4)	0.012	5.34 (0.56–62.1)	0.001
sICAM-1 (ng/ml)	405 (111–1345)	< 0.001	577 (192–1610)	0.009	696 (194–1884)	< 0.001
sVCAM-1 (ng/ml)	397 (19–2252)	< 0.001	847 (101–3230)	0.001	1262 (143–3421)	< 0.001
Coagulation markers:						
Fibrinogen (g/l)	2.99 (1.39–7.19)	0.041	2.75 (1.29–6.99)	0.785	3.11 (1.39–9.00)	0.073
PAI-1 (IU/ml)	1.39 (0.01–17.3)	0.681	1.52 (0.01–18.8)	0.433	0.83 (0.01–17.4)	0.563
Glucose (mmol/l)	5.50 ± 1.10	0.126	5.35 ± 1.26	0.052	4.97 ± 0.29	0.122
eCrCl (ml/min)	79.7 (16.1–157)	0.150	73.5 (15.1–160)	0.790	76.5 (9.58–163)	0.345
Tobacco users, *n* (%)	137 (45.6)	0.410	127 (42.3)	0.082	12 (66.7)	0.071
Alcohol users, *n* (%)	103 (34.3)	0.543	96 (32.0)	0.693	7 (38.9)	0.678

*n*: number of participants; HDL-C: high-density lipoprotein cholesterol; TG: triglycerides; hsIL-6: high-sensitivity interleukin 6; hsCRP: highsensitivity C-reactive protein; sICAM-1: serum intercellular adhesion molecule-1; sVCAM-1: serum vascular cell adhesion molecule-1; PAI-1: plasminogen activator inhibitor-1. Data are expressed as arithmetic mean ± standard deviation, geometric mean (5th and 95th percentile intervals) or % of *n*. ^†^*p*-values between uninfected/infected and uninfected/nadir CD_4_ cell count were obtained with independent *t*-test. ^‡^*p*-values trend was obtained with ANOVA, and for gender, tobacco and alcohol users, Chi-square test was used.

Although the mean levels of hsIL-6, hsCRP, sICAM-1 and sVCAM-1 were the highest in the nadir CD_4_ cell count group, only sVCAM-1 levels differed statistically significantly when compared with the uninfected group. When the low HDL-C and high hsIL-6, hsCRP, sICAM-1 and sVCAM-1 values of the nadir CD_4_ cell count group were compared to the infected participants with a CD_4_ cell count > 200 cells/mm^3^, again only the sVCAM-1 values differed significantly (*p* = 0.046). After adjustments for MAP, alcohol and tobacco use, the overall results did not change. No gender differences were seen.

The odds ratios of the HIV-infected group versus the uninfected group are shown in Table 2. In this study population, having a lower HDL-C level was 3.7 times more likely in the HIV-infected participants. The odds ratio for having higher hsCRP and TG levels, TG:HDL ratio, and hs-IL-6, sICAM-1 and sVCAM-1 levels was respectively, 1.8, 1.7, 3.3, 1.7, 2.0 and 3.9 times more in the HIV-infected participants.

**Table 2.** Odds Ratios Of HIV-Infected Participants vs Uninfected Participants

	*Odds ratios HIV infected vs HIV uninfected*	*95% CI*
HDL-C < 1.36 mmol/l	3.69	2.6–5.2*
TG ≥ 1.0 mmol/l	1.70	1.2–2.3*
TG:HDL ratio ≥ 0.75	3.33	2.4–4.7*
hsCRP ≥ 2.7 mg/l	1.78	1.3–2.5*
hsIL-6 ≥ 4.2 pg/ml	1.67	1.2–2.3*
sICAM-1 ≥ 516 ng/ml	2.04	1.5–2.8*
sVCAM-1 ≥ 693ng/ml	3.92	2.8–5.5*

HDL-C: high-density lipoprotein cholesterol; TG: triglycerides; TG:HDL: triglycerides–high-density lipoprotein ratio; hsCRP: high-sensitivity C-reactive protein; hsIL-6: high-sensitivity interleukin 6; sICAM-1: serum intercellular adhesion molecule-1; sVCAM-1: serum vascular cell adhesion molecule-1. For all variables, the median of total group was used as cut-off value. *Significant.

Partial correlations (adjusted for MAP, tobacco and alcohol use) with a *p*-value < 0.05 are listed in Table 3. HDL-C values correlated inversely with TG levels in all three groups (*r* = –0.23, *p* < 0.001; *r* = –0.16, *p* = 0.005; *r* = –0.55, *p* = 0.41), with log sICAM-1 levels in the uninfected (*r* = –0.14, *p* = 0.02) and infected groups (*r* = –0.15, p = 0.009), and with hsIL-6 levels only in the HIV-infected group (*r* = –0.21, *p* = 0.001). In the nadir CD_4_ cell count group, the CD_4_ cell counts were inversely correlated with hsCRP (*r* = –0.63, *p* = 0.01) and fibrinogen values (*r* = –0.78, *p* = 0.001).

**Table 3.** Partial Correlation Coefficients Between The Different Variables Of The HIV-UNINFECTED, -Infected And Nadir (< 200 Cells/mm^3^) CD_4_ Cell Count Groups

*Variables*	*HIV uninfected (n = 300)*							*HIV infected (n = 300)*						*Nadir CD_4_ cell count (n =18)*			
	*Age*	*cr-PWV*	*HDL-C*	*TG*	*Log hsIL-6*	*Log hsCRP*	*Log sICAM-1*	*Age*	*HDL-C*	*TG*	*Log hsIL-6*	*Log hsCRP*	*Log sICAM-1*	*CD_4_*	*Age*	*HDL-C*	*Log hsCRP*
Age																	
cr-PWV								0.14									
HDL-C																	
TG			–0.23					0.14	–0.16							–0.55	
Log hsIL-6									–0.21								
Log hsCRP					0.45						0.52			–0.63			
Log sICAM-1			–0.14			0.14			–0.15	0.12	0.17	0.14					
Log sVCAM-1							0.27						0.30				
Fibrinogen		–0.12		–0.17	0.29	0.45					0.24	0.33		–0.78			0.58
Log PAI-1				0.17						0.18					–0.67		
Log eCrCl	–0.14							–0.25									

*n*: number of participants; cr-PWV: carotid radialis pulse wave velocity; HDL-C: high-density lipoprotein cholesterol; TG: triglycerides; hsIL-6: high-sensitivity interleukin 6; hsCRP: high-sensitivity C-reactive protein; sICAM-1: serum intercellular adhesion molecule-1; sVCAM-1: serum vascular cell adhesion molecule-1; PAI-1: plasminogen activator inhibitor-1; eCrCl: estimated creatinine clearance. Adjustments were made for mean arterial pressure, tobacco and alcohol use. Only significant (*p* < 0.05) correlation coefficients given.

**Fig. 1.**
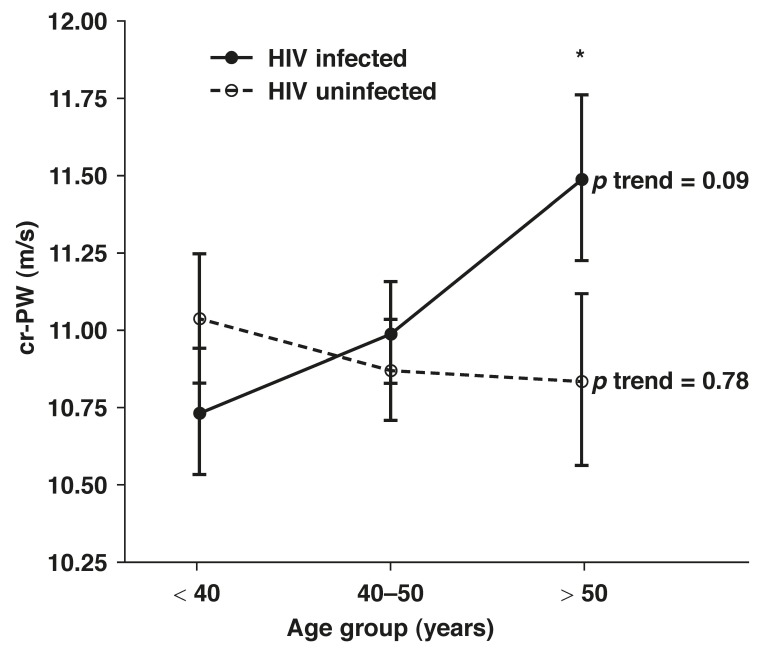
cr-PWV in the HIV-infected and uninfected group with increasing age. Adjusted for gender, BMI, MAP, self-reported alcohol and tobacco use. Values are means ± SEM. *cr-PWV of HIV-infected and uninfected participants differ (*p* = 0.057).

Age correlated positively with cr-PWV values only in the HIV-infected group (*r* = 0.14, *p* = 0.01) after adjusting for MAP as well as tobacco and alcohol use. When the participants were divided into age groups of 10-year intervals, and after adjusting for gender, BMI, MAP, tobacco and alcohol use, a continuous positive trend of increasing cr-PWV levels with age (*p* = 0.09) was detected only in the HIV-infected group (Fig. 1). In the age group older than 50 years, the cr-PWV levels differed between the infected and uninfected groups (*p* = 0.057).

## Discussion

In this case–control study, the HIV-infected participants, who had never received antiretroviral therapy, showed lower HDL-C and higher hsIL-6, hsCRP, sICAM-1 and sVCAM-1 levels than their age-, gender-, BMI- and locality- (urban, rural) matched controls. The higher levels of inflammatory markers and low HDL-C levels could point to endothelial dysfunction, which is seen as the link between infection, inflammation and atherosclerosis.^10^ Furthermore, in the older HIV-infected participants, a positive trend of increasing peripheral cr-PWV was detected, which was not observed in the uninfected participants. This could indicate accelerated vascular aging in these participants. No differences in coagulation factors were detected between the infected and uninfected groups.

The contribution of HIV to endothelial dysfunction is difficult to distinguish from traditional cardiovascular risk factors. Therefore we carefully matched the control participants’ gender, age, BMI and locality to minimise the confounding effect of these conditions on the study findings.

In epidemiological studies, high plasma levels of HDL-C protect against the development of atherosclerosis.^25^ Besides HDL-C’s known ability to promote the efflux of cholesterol from cells in the arterial wall,^26^ and thereby maintaining cell cholesterol homeostasis, HDL-C is also antithrombotic,^27^ and possesses antioxidant and anti-inflammatory properties.^28^,^29^ Our HIV-infected participants had lower serum HDL-C levels and were 3.7 times more likely to have low HDL-C levels than their matched controls, while the lowest levels were seen in the nadir CD_4_ cell count group. The protective effect of HDL-C was lost in our HIV-infected group due to the low levels.

Furthermore, Patel *et al.* demonstrated that triglycerides contribute to atherosclerosis-mediated inflammation by their direct effect on the endothelium and also potentially by attenuating the protective effects of HDL-C against vascular inflammation.^30^ It could therefore be expected that the low serum HDL-C and high TG levels and higher TG/HDL-C ratio seen in the HIV-infected versus uninfected participants worsened the inflammation, as reflected by the higher levels of and odds ratios for having higher levels of hsIL-6 and hsCRP in the infected group. The inverse correlation of HDL-C with hsIL-6 levels (*r* = –0.21, *p* = 0.001), only seen in our HIV-infected group, further emphasises the inflammation in this group. Clinical and epidemiological studies showed that HDL-C concentration is often inversely related with plasma levels of cytokines in atherosclerotic cardiovascular diseases.^31^

While IL-6 is an early stimulator of the inflammatory process and CRP is produced in response to IL-6 secretion, CRP is thought to induce ICAM and VCAM secretion.^32^ These adhesion molecules are known to be expressed in arteries *in vivo* at sites of developing atherosclerosis^33^ and indicate vascular endothelial injury and dysfunction.^3^,^20^,^34^ The CRP-induced expression of endothelial adhesion molecules is inhibited by HDL-C.^31^ The HIV-infected participants in our study showed lower HDL-C and higher hsIL-6, hsCRP, sICAM-1 and sVCAM-1 levels compared to their uninfected controls. The inflammatory process was thereby clearly activated, resulting in endothelial injury. The HIV-infected participants were two and four times more likely to express higher levels of sICAM-1 and sVCAM-1, respectively, further indicating endothelial injury in this group.

It was found that HIV-1 Tat protein (therefore HIV itself) induced the expression of ICAM-1 and VCAM-1 and this could be a possible mechanism by which HIV-1 infection contributes to endothelial injury and accelerated atherosclerosis.^35^,^36^ It could therefore be expected that the HIV-infected, never antiretroviral-treated participants with definite signs of endothelial injury would also show signs of endothelial dysfunction.

Endothelial dysfunction results in increased arterial stiffness, 37 which progresses as arteries become more damaged.^38^ Using femoral PWV, previous studies showed increased aortic (central) stiffness in treated^39^ and untreated^9^ HIV-infected individuals. In our study, the peripheral cr-PWV did not differ between the HIV-infected participants and their age-, gender-, BMI- and locality-matched controls. However, age, although weak, correlated positively with carotid radial PWV (*r* = 0.14, *p* = 0.01) in the HIV-infected group alone.

Age is one of the principal factors modulating PWV,^40^ which is greatest in the elastic aorta and least in muscular arteries such as those of the upper limb.^41^ When divided into 10-year-interval age groups, our HIV-infected participants showed a positive trend of increasing peripheral cr-PWV with age. Since changes in PWV in muscular arteries are not normally found with increasing age,^42^ as in the uninfected group of the present study, this increase in PWV in muscular arteries of the HIV-infected participants was a significant result.

Although increased stiffness could indicate atherosclerosis *per se*, over and above that due to aging,^43^ our results being in muscular arteries may point to premature vascular aging in the HIV-infected participants. This is in agreement with the results of Lorenz *et al*. who obtained a higher ‘vascular’ age of four to five years for HIV-infected patients (treated) compared to controls.^13^ Our results indicate that HIV-1 without the effect of treatment might contribute to accelerated vascular aging and possible early atherosclerosis.

Endothelial dysfunction is also associated with a prothrombotic state,^44^ and studies which investigated the prothrombotic state in HIV-infected populations showed increased levels of PAI-1 activity and fibrinogen.^4^,^45^,^46^ However, in our study neither the PAI-1 activity nor fibrinogen levels were increased in the HIV-infected subjects, indicating no signs of a prothrombotic state. Our findings are in agreement with the findings of James *et al*., who found that HIV infection was not associated with the fibrinogen concentration in Africans.^47^ It is known that black South Africans of African ancestry have high levels of fibrinogen, 48 and it may therefore be that ethnic effects on plasma fibrinogen may have masked the potential effect of HIV infection.

This study has limitations and strengths. It had a case–control design and control participants were carefully matched to the infected participants according to age, gender, BMI and locality. When viewing previous studies regarding HIV and cardiovascular risk, our study population was unique as they were unaware of their infection status and therefore had never received antiretroviral treatment. Therefore, although the evidence of no self-reported diseases was not evaluated, the differences found could probably be attributed to the infection itself and not ARV treatment. Furthermore, although South Africa has the highest infection rate in the world,^7^ data on cardiovascular changes and risk in HIV-1-infected South Africans are limited.

A limitation of the study was that unfortunately, probably due to stigmatisation, which still exists among South African individuals^49^,^50^ and other aspects such as the illness itself and poverty,^51^ the participants did not visit the local clinic or hospital for follow up and CD_4_ cell count determinations. Therefore the sample size of the nadir CD_4_ cell count group was very small and those results should be interpreted with caution. Also, the subjects in this study were newly identified as being HIV infected and therefore the duration of the infection was not known.

The lack of increase in fibrinogen, PAI-1 and cr-PWV levels in the infected group may be related to the possible short duration of the infection, as is also speculated in the study of James *et al*.^47^ This is confirmed by the tendency of increased (although not statistically significant) fibrinogen levels in the nadir CD_4_ cell count group.

A longitudinal study is therefore proposed to further investigate the influence of HIV on the endothelium and prothrombotic state of Africans. A recommendation for future studies would be to perform carotid intima–media thickness measurements to verify endothelial damage and probable atherosclerosis.

## Conclusion

Our findings suggest inflammatory injury of the endothelium, pointing to endothelial dysfunction of never antiretroviral-treated, HIV-1-infected South Africans. Attenuation of the protective effect of HDL-C probably worsened the endothelial inflammation. Although no indication of a prothrombotic state, which could result in atherosclerotic disease could be detected, there was an indication of accelerated vascular aging and probable early atherosclerosis in the older HIV-infected participants.
